# Long non-coding RNA LRRC75A-AS1 facilitates triple negative breast cancer cell proliferation and invasion via functioning as a ceRNA to modulate BAALC

**DOI:** 10.1038/s41419-020-02821-2

**Published:** 2020-08-18

**Authors:** Sijie Li, Di Wu, Hongyao Jia, Zhiru Zhang

**Affiliations:** The Department of Breast Surgery, the First Hospital of Jilin University, 130021 Changchun, China

**Keywords:** Cancer, Breast cancer

## Abstract

As a common female malignancy, triple-negative breast cancer (TNBC) is the most serious subtype in breast cancer (BC). BAALC binder of MAP3K1 and KLF4 (BAALC) is a common oncogene in acute myelocytic leukemia (AML). We sought to explore the role of BAALC in TNBC. In this study, BAALC was significantly upregulated in TNBC tissues and cells. Then, the results of functional assays disclosed that BAALC facilitated cell proliferation, invasion, and epithelial–mesenchymal transition (EMT) processes, but repressed cell apoptosis in TNBC. Next, miR-380–3p was identified as the upstream of BAALC in TNBC cells. Moreover, LRRC75A-AS1 (also named small nucleolar RNA host gene 29: SNHG29) was verified to act as the sponge of miR-380–3p to elevate BAALC expression in TNBC. Besides, LRRC75A-AS1 could negatively regulate miR-380–3p but positively regulate BAALC expression. Finally, rescue assays elucidated that LRRC75A-AS1 facilitated cell proliferation, invasion, and EMT processes in TNBC by targeting miR-380–3p/BAALC pathway. Taken together, our study revealed a novel ceRNA network of LRRC75A-AS1/miR-380–3p/BAALC in accelerating TNBC development, indicating new promising targets for TNBC treatment.

## Introduction

As one of the most common female malignancy, breast cancer (BC) is the major cause of female fatality globally, accounting for around 6.6% of global cancer fatality rate^1^. BC is characterized by high heterogeneity and contains at least five subtypes: HER2 overexpressing, Luminal A, Luminal B, and triple-negative breast cancers (TNBC), which are generally divided into basal-like and claudinlow subtypes^2,3^. TNBC is featured by lacking the expression of estrogen receptors and progesterone receptors^4^. Currently, chemotherapy is the most preferred treatment for TNBC patients^5^. Besides, Chinese herbal compound has been reported to function in BC^6^. Cornus officinalis could inhibit TNBC cell growth^7^. Tanshinone compound extracted from salvia miltiorrhiza was used to treat BC^8^. However, TNBC patients are still confronted with poor clinical outcomes due to high metastatic rate.

Long non-coding RNAs (lncRNAs), a group of non-coding RNAs (ncRNAs) containing more than 200 nucleotides in length, are incapable of encoding proteins. Accumulating evidence has revealed that lncRNAs exert regulatory functions in various cancers, including TNBC. For instance, lncRNA DRHC inhibits cell proliferation in TNBC via down-regulating lncRNA HOTAIR^9^. LncRNA LINC00511 interacts with Snail to facilitate cell growth and invasion in TNBC^10^. LncRNA AWPPH and miRNA-21 interact with each other to modulate TNBC cell proliferation and chemosensitivity^11^. Further, competing endogenous RNA (ceRNA) is a widely reported regulatory network at post transcription level. In ceRNA mechanism, lncRNAs competitively sponge microRNAs (miRNAs) to release miRNAs-targeted mRNAs, therefore modulating the development of cancers including TNBC. For example, lncRNA HEIH modulates TNBC cell proliferation and apoptosis through the miR-4458/SOCS1 axis^12^. LncRNA ADPGK-AS1 facilitates TNBC cell proliferation, migration, and EMT process via modulating the miR-3196/OTX1 axis^13^. Also, lncRNA HCP5 serves as a ceRNA to promote TNBC progression via regulating the miR-219a-5p/BIRC3 axis^14^. A novel lncRNA LRRC75A-AS1 (also named SNHG29) located at 17p11.2 has 892 bp in length. Previously, LRRC75A-AS1 was reported to has oncogenic properties^15^. Besides, LRRC75A-AS1 has been validated in glioblastoma, and it regulates the miR-223–3p/CTNND1 axis to promote glioblastoma through Wnt/β-catenin pathway^16^. In the present study, LRRC75A-AS1 was predicted as the sponge of miR-380–3p, the upstream miRNA of BAALC, and whether the LRRC75A-AS1/miR-380–3p/BAALC axis regulated biological behaviors in TNBC needs further investigation.

Besides, BAALC is a common oncogene in acute myelocytic leukemia (AML)^17–19^. As an example, BAALC played the oncogenic role in AML via ERK pathway^20^. More importantly, BAALC was confirmed to be aberrantly upregulated in TNBC tissues by our studies. Therefore, the supposition was proposed that whether BAALC affected the tumorigenesis of BC, especially TNBC. We sought to examine the function of BAALC in TNBC, and found that LRRC75A-AS1 contributed to TNBC development via functioning as a ceRNA of miR-380–3p to upregulate BAALC, which might provide some novel thoughts for TNBC treatment.

## Materials and methods

### Tissue samples

Total of 62 pairs samples of TNBC tissues and adjacent non-tumor tissues were collected from 62 patients who had not received radiotherapy or chemotherapy before operation. This protocol got approval from the Ethics Committee of the First Hospital of Jilin University, and all participants signed the informed consent forms. Tissue specimens were all frozen in liquid nitrogen right after surgery, and then stored at −80 ^o^C for further analysis.

### Cell culture

Chinese Academy of Sciences (Shanghai, China) provided the TNBC cell lines (MDA-MB-468, MDA-MB-436, MDA-MB-231 and HCC-1937) and normal breast cell line MCF-10A. F15 medium (Hyclone, Logan, UT, USA) was used to cultivate the MDA-MB-231 cells. The F12/DMEM 1:1 medium (Hyclone, Logan, UT, USA) was employed to incubate the MDA-MB-468 or MCF-10A cells. The RPMI-1640 medium was applied to incubate the HCC-1937 cells. The L15/DMEM-H medium was used for incubating the MDA-MB-436 cells. 1% penicillin/streptomycin and 10% fetal bovine serum (FBS) (Gibco, Grand Island, NY, USA) were added to the medium for cultivating with the above cells at 37°C and 5% CO_2_. All cells were authenticated by STR profiling and tested for mycoplasma contamination.

### Cell transfection

Cell transfection was realized by the use of Lip2000 (Invitrogen, Carlsbad, CA, USA) in line with the instructions of manufacturer. GenePharma (GenePharma, Shanghai, China) constructed the shRNAs specific to BAALC (sh-BAALC#1/2) or LRRC75A-AS1 (sh-LRRC75A-AS1#1/2). The nonspecific shRNAs were utilized in control group (sh-NC). The full-length cDNA sequence of BAALC or LRRC75A-AS1 was inserted into pcDNA3.1 overexpression vectors (Invitrogen), with empty vectors as the control. In addition, the miR-380–3p-mimics, miR-380–3p-inhibitor and their corresponding NCs were constructed by Ribobio (Guangzhou, China). Then, 2 μg of above plasmids were transfected into cells. After 48 h of transfection, cells were reaped at third passage. Sequences of above constructs were shown in Table 1.

**Table 1:** Sequences of plasmids used in cell transfection.

shRNA	Sequences (5′-3′)
sh-NC	CCGGAACATCTATTCTAGTTTTTGTCTCGAGAAGATGTTAAGATGTAAATCATTTTTG
sh-BAALC#1	CCGGTTCTACTTATCATGTAAATGTCTCGAGAAGATGAATAGTACATTTACATTTTTG
sh-BAALC#2	CCGGGTCGAAGAATCACAAAGAACTCTCGAGCAGCTTCTTAGTGTTTCTTGATTTTTG
sh-LRRC75A-AS1#1	CCGGTTGCAAATTCGTGAAGAATCACTCGAGAACGTTTAAGCACTTCTTAGTTTTTTG
sh-LRRC75A-AS1#2	CCGGGAGATATATTTGGCAACTTTTCTCGAGCTCTATATAAACCGTTGAAAATTTTTG
**Mimics**	**Sequences (5**′**-3**′**)**
NC mimics	UAGUGUAAUUAACCAUGUCCUU
miR-380–3p-mimics	UAUGUAAUAUGGUCCACAUCUU
NC-inhibitor	AAGAGUGUGACCUAAUACAUUA
miR-380–3p-inhibitor	AAGAUGUGGACCAUAUUACAUA
**pcDNA3.1**	**Sequences (5**′**-3**′**)**
pcDNA3.1/BAALC	GCGCAGGAGGATGGGCTGCGGCGGGAGCCGGGCGGATGCCATCGAGCCCCGCTACTACGAGAGCTGGACCCGGGAGACAGAATCCACCTGGCTCACCTACACCGACTCGGACGCGCCGCCCAGCGCCGCCGCCCCGGACAGCGGCCCCGAAGCGGGCGGCCTGCACTCGGGCATGCTGGAAGATGGACTGCCCTCCAATGGTGTGCCCCGATCTACAGCCCCAGGTGGAATACCCAACCCAGAGAAGAAGACGAACTGTGAGACCCAGTGCCCAAATCCCCAGAGCCTCAGCTCAGGCCCTCTGACCCAGAAACAGAATGGCCTTCAGACCACAGAGGCTAAAAGAGATGCTAAGAGAATGCCTGCAAAAGAAGTCACCATTAATGTAACAGATAGCATCCAACAGATGGACAGAAGTCGAAGAATCACAAAGAACTGTGTCAACTAG
pcDNA3.1/LRRC75A-AS1	GGATTGGGATATTCCGACTCCTTAAGGGCCTGGCGCACATAAGGTGTGACCTTTTCATTCCCGTTGTTATGGAGGGCCACATCTGCCAGAGCCTGGAGTCTGCGAAGGCCGGGACCCGGTTCCCCGGCCCACAGTGGGGGTGTGCAAACCCGAGAGAACTGGGTTGCAAATTCGTGAAGAATCAGCATCATGTTTGGCAGCTGAGTATTGGAGCCAGGAGCCTGCCATGAGGTTATATTCCCAGAGGATGTCAGTCCCAAGGACCAGTAGCTGCCATCAGTTTGGATTCTGAAAACTAACTGGCATCAACACTGGGTGTAGAAACATGCTTGCCTTATGTATCAGAGGACATGCTCAGCAGATCCAAGAGATATATTTGGCAACTTTTTCTAGAAAAGGCACATTGGGTATCATTCATTACATTCTTGAGTTTTTTTGGGTTTTTTTTTTTTTTTTTGAGACAGTCTTGCTGTATTGCCCAGGCTGGAGTGTGGTGGCACAATCACAGCTCATTGCATCCTCAATCACCCAGGCCTAAGCAATCCTCCCACCTTGTAGCTGGGACTACAGCTCACAGCACACCTGGCTAAAATTTTTTTTTTGTTGAGACGGATTCTCTATGTTGCCCAGGCTGGTCTCAGGCTCCTGGGCTCAGATGGTCCTCCTGCCTCAGCTTCCAAAGGCACAGGCCAAGTTGTAGCTTTGTCCCTTGCCATCATGCCCAACAAGAGGTTCTATACCTTTTAATGAATTGACTTTCATAAATTGGTTATGTTGGTGGGCAAAGTTCTTTAAGCTGGAAATTGTAAATTCCTCCTGAAATGTTTTTTCATGCAGTTACCATGAACTAATACTACAATAAAGGATGGTCTTGGGTGTCAATTCTTGAAAA

### Quantitative RT-PCR

Trizol reagent (Invitrogen, Carlsbad, CA) was used to extract the total RNA in line with the protocol of supplier. With regard to lncRNAs or mRNAs, SuperScriptTM II reverse transcriptase (Invitrogen, Carlsbad, CA) was used to take the reaction of reverse transcription. As for the reverse transcription of miRNAs, a miScript reverse transcription kit (Qiagen, Hilden, Germany) was applied. qRT-PCR was conducted by the use of SYBR@ Premix Ex TaqTM (TaKaRa Bio Group, Shiga, Japan). Relative expression normalizing to GAPDH or U6 was measured via 2^-∆∆Ct^ method.

### Colony formation assay

After 48 h of transfection, cells were collected for colony formation assay. Cells at the concentration of 1 × 10^3^ each well were cultivated in 6‐well plates at 37 °C for 14 days. After that, PBS was used to rinse the cells for two times. Then, 4% formaldehyde and GIMSA were used to fix and stain the cells for 15 min, separately. At last, the colony formation ability was evaluated by counting the number of stained colonies.

### CCK-8 assay

The CCK-8 solution was procured from Dojindo (Osaka, Japan) to undertake cell proliferation assay. After 48 h of transfection, MDA-MB-468 and MDA-MB-436 cells were seeded into the 96-well plates at the concentration of 5000 cells per well, followed by addition of 10 μl CCK-8 solution for 2 h in 5% CO_2_ at 37^o^C. Cell proliferation was detected at indicated times via measuring the absorbance at 450 nm with spectrophotometer (Thermo Fisher Scientific, Waltham, MA, USA).

### Cell apoptosis

The Annexin V-FITC Apoptosis Detection Kit (JingMei Biotech, Beijing, China) was acquired to estimate cell apoptosis rate in line with instructions of manufacturer. Simply put, the transfected cells were collected after 48 h and seeded into the 6-well plates at the concentration of 1 × 10^3^ cells in each well. Thereafter, 5 μL Annexin V-FITC and 10 μL propidium iodide (PI) (20 μg/mL) were put into 100 μL cell suspension for cultivating at 37°C. The whole process took 15 min in the dark room. After that, a FACSCaliber flow cytometry (BD Biosciences, Franklin Lakes, NJ, USA) was used to analyze the rate of apoptotic cells.

### Relative caspase activity determination

On the basis of the protocols, the activities of caspase-3/8/9 were determined by use of Caspase-3 Activity Kit, Caspase-8 Activity Kit, and Caspase-9 Activity Kit, respectively. Simply put, after 48 h of incubation, the lysates of MDA-MB-468 and MDA-MB-436 cells (5 × 10^6^) were subjected to centrifugation and collection. Afterwards, 10 μl protein in the cell lysates was supplemented into 96-well plates, followed by the adding of 80 μl reaction buffer that included caspase substrate (2 mM). Microplate reader was used to detect the caspase activities at the absorbance of 405 nm after they were incubated at 37 °C for 5 h.

### Transwell assay

The transwell plates (24-well, 8μm pore membrane; Corning, NY, USA) pre-incubated with Matrigel (Corning, NY, USA) were employed to estimate the invasive abilities of cells. The upper layer of the chamber was inoculated with 2 × 10^4^ cells which were suspended in serum-free medium. The lower layer of the chamber was used to cultivate the complete culture medium with 20% FBS. Twenty-four hours later, 4% formaldehyde and crystal violet were respectively used to fix and stain the cells which crossed the membranes, followed by observing and imaging cells under a Nikon microscope. The cells remained in the upper chamber were cleared by a swab.

### JC-1 assay

JC-1 assay is a method to detect mitochondrial membrane potential. The decrease of mitochondrial electrochemical potential gradient is a marker of early apoptosis. The transformation of from red fluorescence to green fluorescence can be used as an indicator of early apoptosis. For measuring the mitochondrial transmembrane potential (ΔΨm), JC-1 assay was undertaken as instructed by provider (Beyotime, Shanghai, China). In short, after being washed for two times, cells were resuspended in PBS which included 0.1 μM JC-1 monomer. Then the cells were cultivated at the temperature of 37 °C for 15 min. Cold PBS was used to rinse the cells. Thereafter, a FACSort flow cytometer (Becton Dickinson) which equipped with a 488-nm argon laser and three fluorescence detectors was used to record the cell fluorescence. JC-1 fluorescence in the FL1 and FL2 channels were analyzed for detecting the form of liquid crystal and the fluorescence of dye monomer. Green fluorescence: the monomer, red fluorescence: the J-aggregates, orange fluorescence: merged photo.

### RNA immunoprecipitation (RIP)

NP-40 lysis buffer, which was added with 1 mM dithiothreitol, 1 mM phenylmethylsulfonyl fluoride (PMSF), RNase inhibitor (200 U/ml) (Life Technologies) and 1% protease inhibitor cocktail (Sigma-Aldrich), was used to lyse 1 × 10^7^ cells collected after centrifugation. In order to produce the antibody-coated beads, NT2 buffer (50 mM tris-HCl, 150 mM NaCl, 1 mM MgCl2, and 0.5% NP-40) was used to rinse the protein G Sepharose 4 Fast Flow bead slurry (GE Healthcare) and then the beads were cultivated with antibody against Ago2 or IgG (the negative control). As for RIP, the antibody-coated Sepharose beads were used to cultivate the cell lysates overnight. Beads were washed by the use of cold NT2 buffer, followed by the cultivation with proteinase K (10 mg/ml) (Sigma-Aldrich). The RNeasy Mini Kit (Qiagen) was used to purify the RNAs bound to beads and then determined by qRT-PCR.

### Subcellular fractionation

A RiboTrap Kit (MBL International) was employed to carry out the cellular fractionation in MDA-MB-468 and MDA-MB-436 cells (1 × 10^6^) in line with the instructions of supplier. Then, RNA in nucleus and cytoplasm was separated, followed by detection of LRRC75A-AS1 via qRT-PCR. Meanwhile, GAPDH and U6 were served as the cytoplasmic or nuclear control, respectively.

### FISH assay

On the basis of the manufacturer’s guide, FISH kit (Guangzhou Biosense Bioscience Co., Ltd, Guangzhou, China) was adopted to carry out the RNA FISH assays for observing the subcellular localization of LRRC75A-AS1 in MDA-MB-468 and MDA-MB-436 cells (1 × 10^6^). BersinBio Co. LTD (Guangzhou, China) composed and designed the Cy3-labeled LRRC75A-AS1 probes. Simply put, 4% formaldehyde was applied to fix MDA-MB-468 and MDA-MB-436 cells at room temperature (RT) for 15 min. Then, 0.5% Triton X-100 was used to permeabilize the fixed cells. After that, probes were used to cultivate the cells overnight at 42 °C. DAPI was used to stain the cell nuclei at RT for 5 min. Fluorescence images were acquired by the use of a fluorescence microscope (Leica, Hilden, Germany).

### Immunofluorescence

Firstly, 10% formaldehyde was used to fix the collected cells (2.5 × 10^4^/well) in 6-well plates for 10 min. Then the cells were blockaded with 10% FBS, followed by permeabilization via 0.5% Triton X-100. Then the cells were cultivated with the Ki67 antibody (1:10) and DAPI (1:100) staining at 25 °C for 30 min in a dark room. A fluorescence microscope using a ×20 objective was used to analyze the smears.

### Western blot analysis

Before the proteins were isolated via RIPA lysis buffer with protease inhibitors, cells (5 × 10^5^) were rinsed in 6-well plates by the use of PBS for two times. Then, the isolated proteins were separated through SDS/PAGE gel and then shifted to the PVDF membranes, which were later processed with primary and secondary antibodies. Primary antibodies against BAALC (ab251772), E-cadherin (ab53226), N-cadherin (ab18203), Slug (ab106077), Twist (ab49254), and the loading control GAPDH (ab9485), as well as the secondary antibody horseradish peroxidase-linked goat anti-rabbit IgG (ab205718), were all from Abcam (Cambridge, UK). Finally, the proteins were observed via ECL system (Santa Cruz Biotechnology, Santa Cruz, CA).

### Luciferase reporter assay

The 96-well plate was used to seed the cells (2 × 10^4^ cells/well) for co-transfecting with miR-380–3p or either empty vector and luciferase reporter which included the mutant type or wild type LRRC75A-AS1 fragment by the use of Lipofectamine 2000 (Invitrogen). The mutated type or wild type BAALC fragment was also inserted into luciferase reporter vector and then co-transfected with indicated transfection plasmids for luciferase assay. Dual‐Luciferase reporter assay system (Promega, USA) was used to detect the activities after the 48 h transfections. Renilla luciferase activity was served as the standard of firefly luciferase activity, so as to explicate the differences in the efficiency of transfection.

### RNA pull‐down assay

RNA pull down assay was studied using 1 × 10^7^ MDA-MB-468 and MDA-MB-436 cells. DNA templates with biotin-UTP, NTP mix and T7 RNA polymerase (Promega) were used to transcribe the biotin-labeled RNAs which were handled with RNase-free DNase I (Promega) and purified by using RNeasy Mini kit (QIAGEN). Then cell lysates were resuspended in the RIP buffer, followed by cultivation with the biotinylated RNA probes at the temperature of 37 °C for 1 h. The non-biotin-labeled RNA probes were seen as the negative control. Subsequently, the binding reaction was added with 60 ul of streptavidin agarose beads (Invitrogen) which were cultivated for 1 h at 37 °C afterwards. After being purified, the RNAs pulled down in each group were analyzed via qRT-PCR.

### Statistical analysis

Statistical data analysis was carried out by the use of SPSS 17.0. Mean ± SD was used to present data (all conformed to normal distribution). Two-tailed Student’s t test was used to take the comparison between two groups, while one-way ANOVA applied for that among no less than two groups. The overall survival was examined using the Kaplan–Meier analysis method. When the value of *P* was less than 0.05, the differences were supposed to be significant statistically.

## Results

### BAALC regulated TNBC cell proliferation, apoptosis, invasion, and EMT processes

**Fig. 1:**
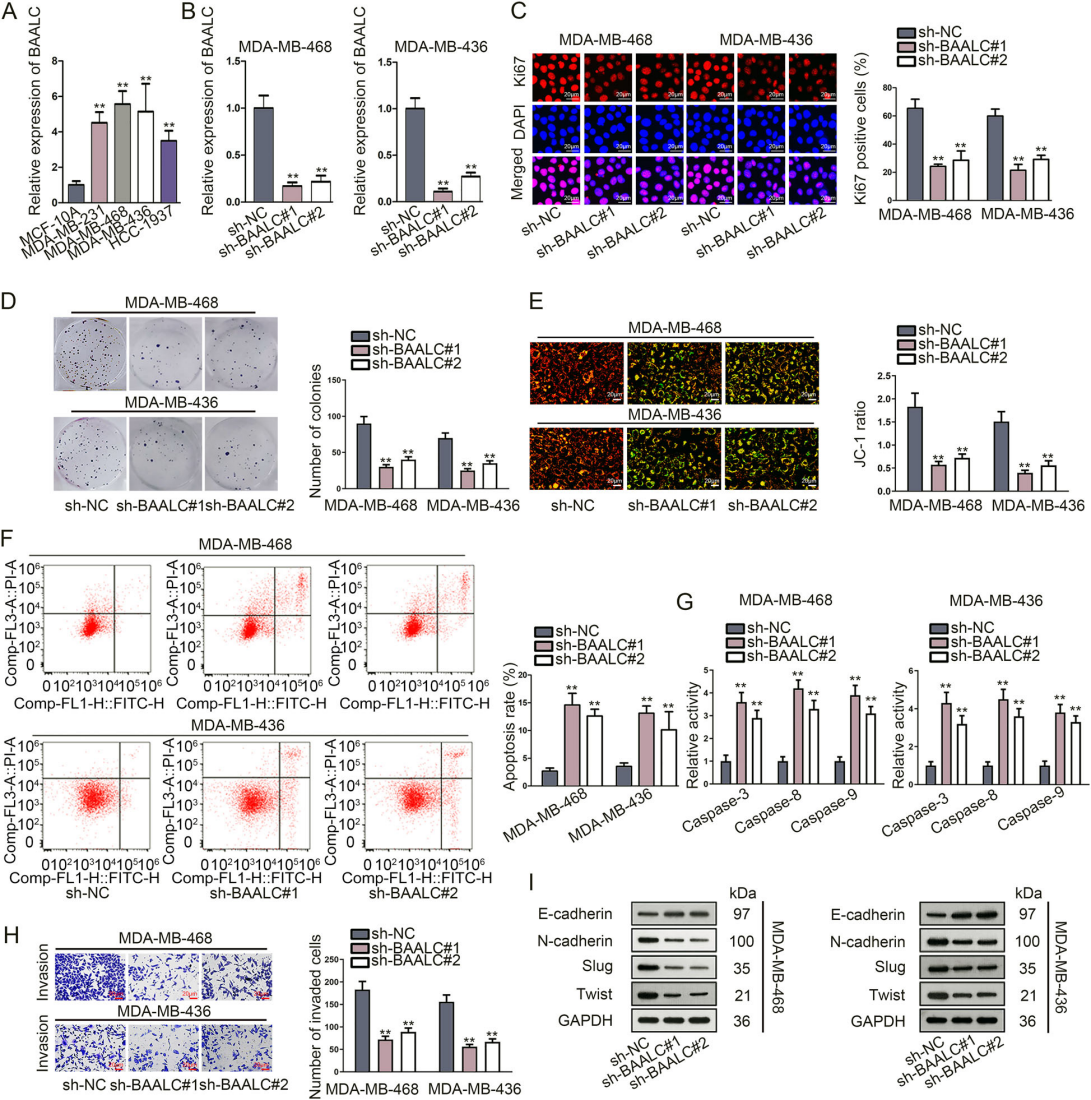
BAALC regulated TNBC cell proliferation, apoptosis, invasion and EMT. **a** qRT-PCR was carried out to examine the expression level of BAALC in TNBC cells (MDA-MB-231, MDA-MB-468, MDA-MB-436, and HCC-1937) and normal mammary epithelial cells (MCF-10A). **b** qRT-PCR and western blot verified BAALC knockdown efficiency in sh-BAALC#1/2 transfected MDA-MB-468 and MDA-MB-436 cells. **c**–**d** Ki67 immunofluorescence staining (scale bar = 50 μm) and colony formation assays detected the proliferation ability of TNBC cells when down-regulating BAALC. **e** Mitochondrial membrane potential was measured by JC-1 assay in sh-BAALC#1/2 transfected cells (scale bar = 200 μm). **f** Flow cytometry analysis evaluated cell apoptosis rate in response to BAALC depletion. **g** Caspase-3/8/9 activities were measured in sh-BAALC#1/2 transfected cells. **h** Transwell assay assessed cell invasion before or after knocking down BAALC (scale bar = 200μm). **i** Western blot detected the expression of EMT-related proteins in TNBC cells transfected with sh-BAALC#1/2. Error bars represent the mean ± SD of at least three independent experiments. ^**^P < 0.01.

To explore the role of BAALC in TNBC, firstly, qRT-PCR analysis confirmed that BAALC was an abnormally upregulated gene in TNBC tissues compared with paired controls (Fig. S1A). Meanwhile, Kaplan–Meier analysis illustrated that high BAALC expression was closely correlated with the poor prognosis of TNBC patients (Fig. S1B). Additionally, the expression level of BAALC in TNBC cells (MDA-MB-231, MDA-MB-468, MDA-MB-436 and HCC-1937) and normal mammary epithelial cells (MCF-10A) was also measured by qRT-PCR and western blot. The result revealed that BAALC was notably upregulated in TNBC cells in comparison with MCF-10A cells (Fig. 1a and S1C). More importantly, the function between BAALC-lowly-expressed MCF-10A and two TNBC cells (MDA-MB-468 and MDA-MB-436) with high BAALC expression was compared. The outcomes of CCK-8, Ki67 immunofluorescence staining and colony formation assays demonstrated that the proliferation ability of MDA-MB-468 and MDA-MB-436 cells was much stronger than MCF-10A cells (Fig. S1D–F). In contrast, JC-1 assay, flow cytometry analysis and caspase 3/8/9 activity detection assay measured that the apoptosis ability of MDA-MB-468 and MDA-MB-436 cells was weaker than MCF-10A cells (Fig. S1G–I). Moreover, transwell assay detected that MDA-MB-468 and MDA-MB-436 cells were more prone to invasion than MCF-10A cells (Fig. S1J). Meanwhile, western blot disclosed that MDA-MB-468 and MDA-MB-436 cells possessed less E-cadherin but more N-cadherin, slug and twist than MCF-10A cells (Fig. S1K). Overall, MDA-MB-468 and MDA-MB-436 cells with higher BAALC expression possessed much malignant behaviors than MCF-10A cells with lower BAALC level.

To evaluate the regulatory function of BAALC in TNBC, qRT-PCR and western blot assessed the knockdown efficiency of BAALC in sh-BAALC#1/2 transfected MDA-MB-468 and MDA-MB-436 cells. As expected, BAALC expression was observably decreased by sh-BAALC#1/2 (Fig. 1b and S1L). Next, a range of functional assays were performed. It was detected that both the percentage of Ki-67 positive cells and the number of colonies were notably declined upon BAALC deficiency (Fig. 1c, d). Further, we also observed a marked inhibition on cell viability in response to BAALC downregulation (Fig. 1M). Then, mitochondrial membrane potential, whose change could indicate early apoptosis, was analyzed by JC-1 fluorescent probe^21^. Results displayed that JC-1 ratio of TNBC cells was decreased by knockdown of BAALC (Fig. 1e). Flow cytometry analysis verified that cell apoptosis rate was enhanced under BAALC suppression (Fig. 1f). Also, the activity of apoptosis-associated proteins (caspase-3, caspase-8, and caspase-9) was elevated in cells transfected with sh-BAALC#1/2 (Fig. 1g), further suggesting that BAALC depletion stimulated cell apoptosis. Moreover, transwell assay disclosed that number of invaded cells was obviously reduced in face of BAALC silence (Fig. 1h). Consistently, the expression of E-cadherin was elevated while that of N-cadherin, slug and twist was reduced after knocking down BAALC (Fig. 1i), indicating hindered EMT resulting from BAALC inhibition. To sum up, BAALC facilitated cell proliferation, invasion and EMT, yet inhibited cell apoptosis in TNBC.

### MiR-380–3p targeted BAALC and negatively regulated BAALC expression

**Fig. 2:**
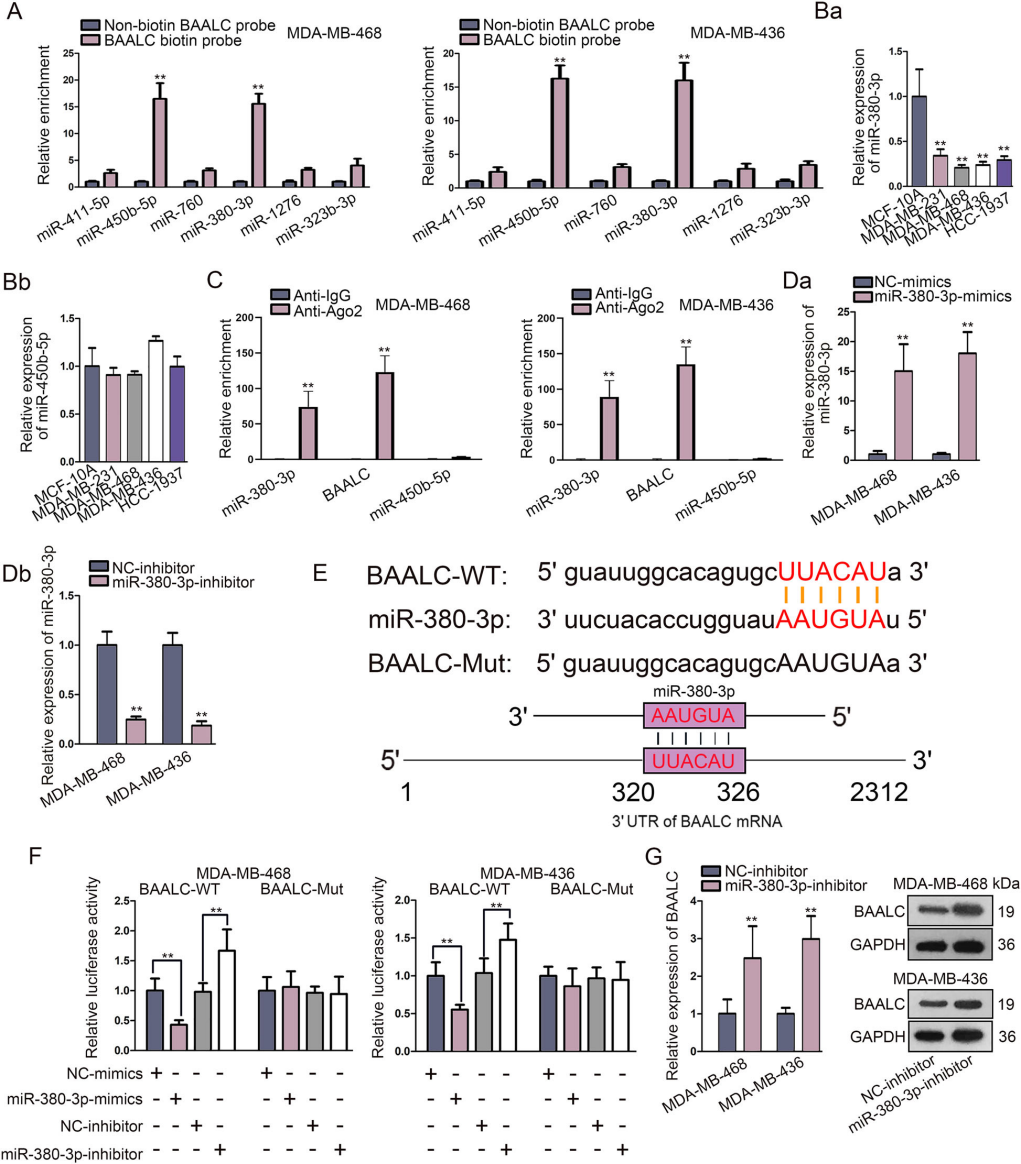
MiR-380–3p targeted BAALC and negatively regulated BAALC expression. **a** RNA pull down assay examined the enrichment of candidate miRNAs in biotin-labeled BAALC group. **ba**, **bb** qRT-PCR detected miR-380–3p and miR-450b-5p expression in TNBC cells and MCF-10A cells. **c** RIP assay detected the enrichment of miR-380–3p, BAALC and miR-450b-5p in anti-Ago2 and anti-IgG groups. **da**, **db** qRT-PCR verified the knockdown or overexpression efficiency of miR-380–3p in miR-380–3p-mimics or miR-380–3p-inhibitor transfected cells. **e** Sequences of wild type/mutant BAALC and miR-380–3p with or without interaction were obtained via starBase prediction (upper); schematic diagram exhibited the interaction between miR-380–3p and BAALC (lower). **f** Luciferase reporter assay detected the luciferase activity of wild and mutant BAALC under the condition of miR-380–3p knockdown or overexpression. **g** qRT-PCR and western blot revealed the influence of miR-380–3p downregulation on mRNA and protein expressions of BAALC in TNBC cells. Error bars represent the mean ± SD of at least three independent experiments. ^**^P < 0.01.

Since miRNAs were reported to target mRNAs and regulate their expression in many diseases^22–24^, we subsequently searched for the upstream miRNAs of BAALC in TNBC. By utilization of starBase database^25^, six potential miRNAs (miR-411–5p, miR-450b-5p, miR-760, miR-380–3p, miR-1276, and miR-323b-3p) were identified. According to the results of RNA pull down assay, only miR-380–3p and miR-450b-5p among above 6 candidates were notably pulled down by BAALC biotin probe rather than non-biotin BAALC probe (Fig. 2a), suggesting that miR-380–3p and miR-450b-5p were more likely to bind with BAALC in TNBC. For further screening, the expression of miR-380–3p and miR-450b-5p in TNBC cells and MCF-10A cells was detected. As a result, we found that miR-380–3p was notably downregulated in TNBC cells compared to MCF-10A cells, while the expression of miR-450b-5p was not statistically different in these cells (Fig. 2ba, bb). Further, RIP assay revealed that only miR-380–3p and BAALC were significantly enriched in anti-Ago2 groups relative to anti-IgG groups, while no evident miR-450b-5p signals were observed in both groups (Fig. 2c). Subsequently, the overexpression or knockdown efficiency of miR-380–3p was verified by qRT-PCR in miR-380–3p-mimics or miR-380–3p-inhibitor transfected cells (Fig. 2da, db). Figure 2e revealed the binding site between wild type/mutant BAALC and miR-380–3p (upper); meanwhile, the schematic diagram exhibited the interactions between miR-380–3p and BAALC (lower). The effectiveness of the indicated binding site was verified by luciferase reporter assays. MiR-380–3p overexpression significantly reduced, while knockdown of miR-380–3p overtly enhanced, the luciferase activity of wild type BAALC. Nevertheless, the luciferase activity of mutant BAALC kept unchanged under the same conditions (Fig. 2f). Moreover, the regulatory role of miR-380–3p in BAALC expression was demonstrated. Results indicated that inhibition of miR-380–3p significantly elevated the expression of BAALC at both mRNA and protein levels in TNBC cells (Fig. 2g), whereas miR-380–3p overexpression remarkably downregulated both above levels of BAALC in these two cells (Fig. S2A). To sum up, miR-380–3p could bind with BAALC and negatively regulate BAALC expression in TNBC cells.

### LncRNA LRRC75A-AS1 sponged miR-380–3p and negatively modulated miR-380–3p expression

**Fig. 3:**
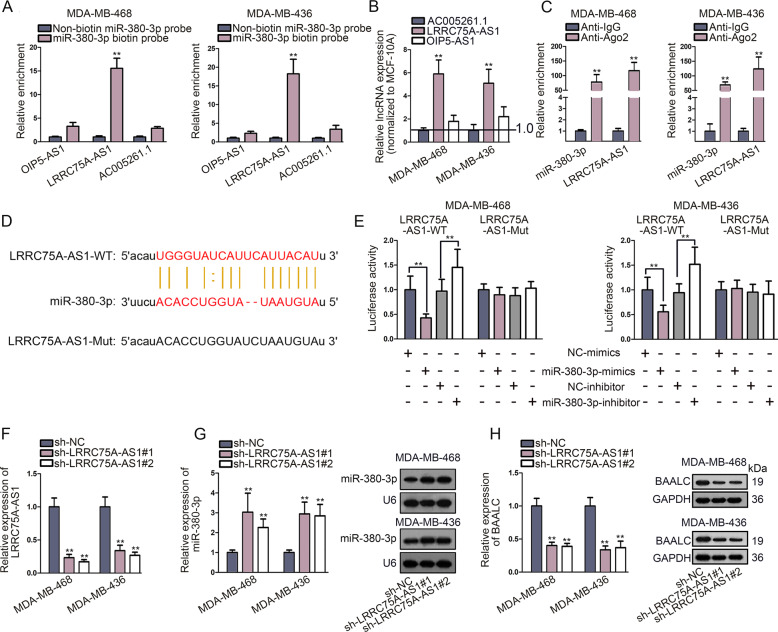
LncRNA LRRC75A-AS1 sponged miR-380–3p and negatively modulated miR-380–3p expression. **a** RNA pull down assay detected the enrichment of OIP5-AS1, LRRC75A-AS1 and AC005261.1 in biotinylated miR-380–3p group. **b** qRT-PCR examined the expression of OIP5-AS1, LRRC75A-AS1, and AC005261.1 in TNBC cells and MCF-10A cells. **c** RIP measured the enrichment of LRRC75A-AS1 and miR-380–3p in anti-Ago2 or anti-IgG group. **d** Sequences of wild/mutant LRRC75A-AS1 and miR-380–3p were obtained according to the prediction of starBase. **e** Luciferase reporter assay detected the luciferase activity of wild or mutant type LRRC75A-AS1 under the condition of miR-380–3p knockdown or overexpression. **f** Knockdown efficiency of LRRC75A-AS1 was verified by qRT-PCR. **g**–**h** Influence of LRRC75A-AS1 knockdown on the expression of miR-380–3p and BAALC in TNBC cells was examined through qRT-PCR, norther blot (U6 served as the internal control) and western blot (GAPDH served as the internal control). Error bars represent the mean ± SD of at least three independent experiments. ^**^P < 0.01.

After confirming miR-380–3p as the upstream miRNA of BAALC, we next sought to find out the upstream lncRNA sponging miR-380–3p in TNBC. Under the screening conditions (strict stringency of CLIP data; at least four cancer types in Pan-Cancer), OIP5-AS1, LRRC75A-AS1, and AC005261.1 were identified as the potential sponges of miR-380–3p. Afterwards, RNA pull down assay measured that only LRRC75A-AS1 was significantly pulled down by biotinylated miR-380–3p in TNBC cells (Fig. 3a). Also, qRT-PCR analysis revealed that only the expression of LRRC75A-AS1 was significantly upregulated in TNBC cells compared with MCF-10A cells (Fig. 3b). Therefore, LRRC75A-AS1 was selected to do the following experiments. Additionally, RIP assay detected that miR-380–3p and LRRC75A-AS1 were both enriched in anti-Ago2 group (Fig. 3c). Moreover, it also confirmed that LRRC75A-AS1 was significantly upregulated in TNBC tissues compared with non-tumor tissues (Fig. S2B). Of note, higher LRRC75A-AS1 level could led to shorter overall survival time of TNBC patients (Fig. S2C). Further, subcellular fraction and FISH assays were performed to analyze the distribution of LRRC75A-AS1 in TNBC cells. Interestingly, we disclosed that LRRC75A-AS1 was mainly distributed in the cytoplasm (Fig. S2D–E). Besides, according to lncLocator analysis^26^, LRRC75A-AS1 got score of 0.190381739971 in cytoplasm, 0.0428120358959 in nucleus, 0.0841591539673 in ribosome, 0.656998637521 in cytosol and 0.0256484326448 in exosome. All these data indicated that LRRC75A-AS1 exerted the regulatory function at post-transcriptional level in TNBC cells.

Then, the binding sites between wild type/mutant LRRC75A-AS1 and miR-380–3p were predicted from starBase (Fig. 3d). Subsequently, luciferase report assay indicated that the luciferase activity of wild type LRRC75A-AS1 was significantly reduced by miR-380–3p overexpression but markedly elevated under miR-380–3p downregulation, whereas that of mutant LRRC75A-AS1 presented no significant changes in response to miR-380–3p overexpression or downregulation (Fig. 3e). So far, LRRC75A-AS1 was validated as the miR-380–3p sponge in TNBC. Moreover, TNBC cells were transfected with sh-LRRC75A-AS1#1/2 to measure the knockdown efficiency of LRRC75A-AS1 (Fig. 3f). It was proved that knockdown of LRRC75A-AS1 notably enhanced the expression of miR-380–3p and decreased that of BAALC at both mRNA and protein levels (Fig. 3g, h). In addition, the levels of BAALC mRNA and protein were both strengthened in pcDNA3.1-LRRC75A-AS1 transfected cells (Fig. S2F). Further, RIP assay detected the fortified enrichment of miR-380–3p and BAALC in TNBC cells with silenced LRRC75A-AS1 (Fig. S2G). Afterwards, qRT-PCR and western blot measured that co-treatment of anti-DICER could reverse the inhibitory impact of LRRC75A-AS1 depletion on BAALC mRNA and protein levels (Fig. S2H), further indicating LRRC75A-AS1 was the ceRNA of BAALC. Moreover, co-transfection of miR-380–3p inhibitor or pcDNA3.1-BAALC rescued the suppressed BAALC expression in sh-LRRC75A-AS1 transfected cells (Fig. S2I). In a summary, LRRC75A-AS1 sponged miR-380–3p to upregulate BAALC expression in TNBC cells.

### LRRC75A-AS1 facilitated the malignant phenotypes of TNBC cells by sponging miR-380–3p and up-regulating BAALC

**Fig. 4:**
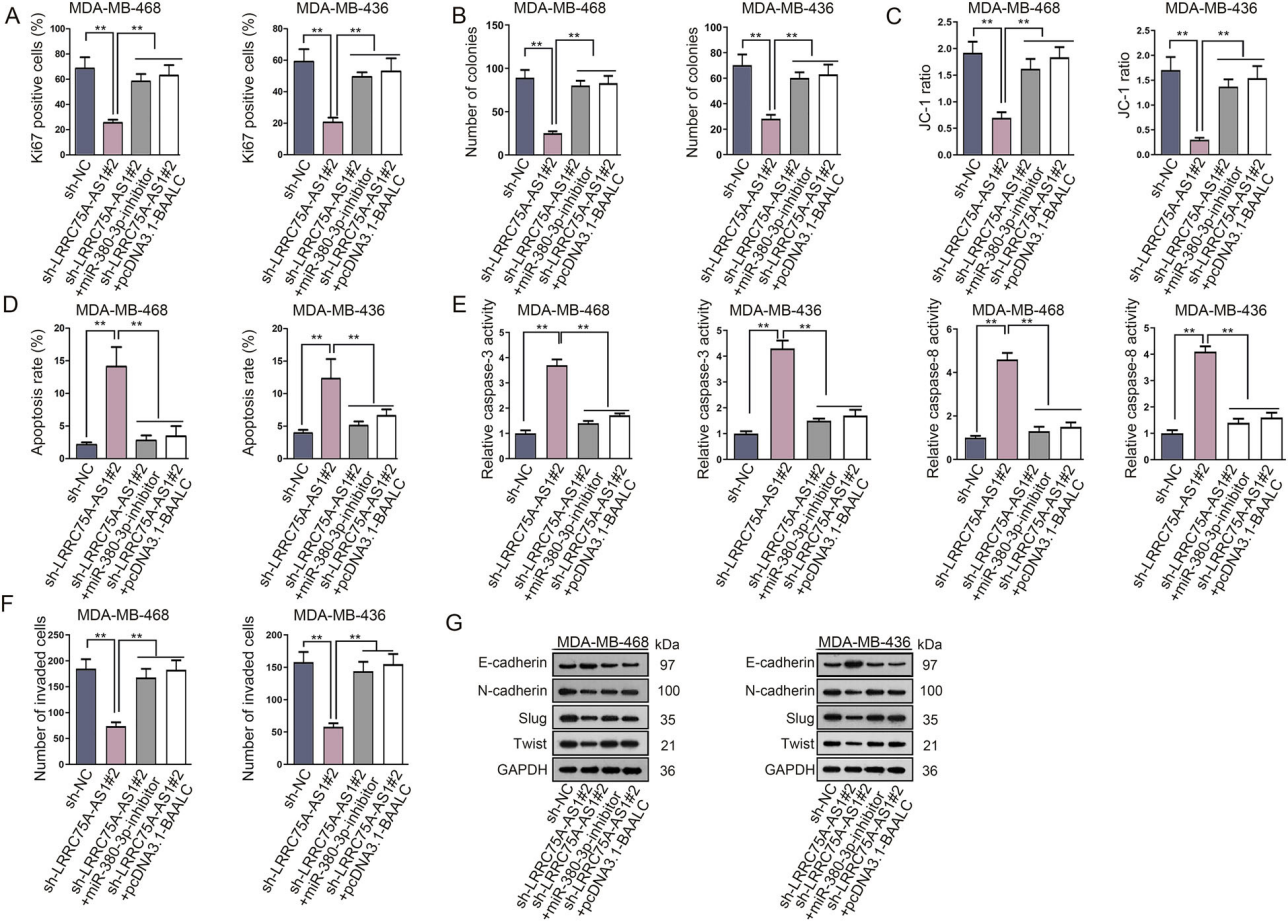
LRRC75A-AS1 modulated TNBC cell proliferation, apoptosis, invasion and EMT by sponging miR-380–3p and up-regulating BAALC. **a**, **b** Ki67 Immunofluorescence staining and colony formation assays detected the proliferation ability of TNBC cells transfected with sh-LRRC75A-AS1#2 or co-transfected with sh-LRRC75A-AS1#2+miR-380–3p-inhibitor/pcDNA3.1-BAALC. **c** JC-1 assay measured the effect of miR-380–3p-inhibitor or pcDNA3.1-BAALC on JC-1 ratio in TNBC cells transfected with sh-LRRC75A-AS1#2. **d** Flow cytometry assay examined the impact of miR-380–3p-inhibitor or pcDNA3.1-BAALC on the apoptosis rate of TNBC cells transfected with sh-LRRC75A-AS1#2. **e** Protein activity detection examined the influence of miR-380–3p-inhibitor or pcDNA3.1-BAALC on caspase-3/8 activity in TNBC cells transfected with sh-LRRC75A-AS1#2. **f**, **g** Transwell and western blot examined the effect of miR-380–3p-inhibitor or pcDNA3.1-BAALC on the invasion and EMT in TNBC cells transfected with sh-LRRC75A-AS1#2. Error bars represent the mean ± SD of at least three independent experiments. ^**^P < 0.01.

After verifying the function of LRRC75A-AS1/miR-380–3p/BAALC network in TNBC, we then conducted rescue assays. As revealed by Ki67 immunofluorescence staining and colony formation assays, TNBC cell proliferation ability was inhibited by LRRC75A-AS1 knockdown, but was then remedied by miR-380–3p downregulation or BAALC overexpression (Fig. 4a, b). Also, JC-1 assay measured that miR-380–3p-inhibitor or pcDNA3.1-BAALC offset the repressing effects of LRRC75A-AS1 depletion on JC-1 ratio (Fig. 4c). Flow cytometry assay revealed that the promoting influence of LRRC75A-AS1 knockdown on TNBC cell apoptosis was counteracted by miR-380–3p downregulation or BAALC overexpression (Fig. 4d). Additionally, miR-380–3p inhibition or BAALC up-regulation reversed the enhancing impact of LRRC75A-AS1 knockdown in caspase-3/8 activity (Fig. 4e). Further, it manifested that co-transfection of miR-380–3p-inhibitor or pcDNA3.1-BAALC obviously rescued the suppression of LRRC75A-AS1 knockdown on TNBC cell invasion and EMT processes (Fig. 4f, g). In a word, LRRC75A-AS1 sponged miR-380–3p to upregulate BAALC, so as to facilitate the development of TNBC.

## Discussion

BAALC is recognized as an upregulated gene in AML. Also, according to a previous study, BAALC could interact with a scaffold protein MEK kinase-1 (MEKK1) and Krüppel-like factor 4 (KLF4) to activate oncogenic ERK pathway^20^. Nonetheless, the function of BAALC in TNBC remained unknown. In this research, BAALC was upregulated in TNBC tissues and cells, indicating the research value of BAALC in TNBC. Further, BAALC downregulation suppressed cell proliferation, invasion and EMT, but contributed to cell apoptosis in TNBC, suggesting the tumor-facilitating role of BAALC in TNBC.

Then, bioinformatics analyses were used to find out the miRNA upstream of BAALC in TNBC. More importantly, the function of miRNA in BC has been revealed by various studies. For example, miRNA-589 functions as a tumor inhibitor via directly targeting metastasis-associated protein 2 (MTA2) in BC^27^. MiR-29b-3p promotes the proliferation and migration of MDA-MB-231 cells (TNBC cells) via down-regulating TRAF3^28^. MiRNA-5195–3p elevates the chemosensitivity of TNBC to paclitaxel via suppressing EIF4A2^29^. In the present study, miR-380–3p was validated to target BAALC in TNBC cells. Moreover, miR-380–3p could negatively regulate BAALC in TNBC. Currently, the reports about the function of miR-380–3p in cancers including TNBC are limited. According to previous findings, miR-380–3p regulated by Nrf2 suppressed cell proliferation and strengthened the PQ-induced toxicity in mouse neuroblastoma cells via obstructing Sp3 mRNA translation^30^. MiR-380–3p could modulate melanogenesis via targeting SOX6 in melanocytes^31^. Herein, our research firstly revealed the anti-tumor function of miR-380–3p in TNBC.

Recently, the existence of ceRNA mechanism has been verified in various diseases according to various reports. For instance, lncRNA GSTM3TV2 enhances the expression of LAT2 and OLR1 via competitively sponging let-7 to facilitate gemcitabine resistance in pancreatic cancer^32^. LncRNA HOTTIP elevates the expression of CCL3 and stimulates cartilage degradation through sponging miR-455–3p^33^. Therefore, whether ceRNA network involving miR-380–3p and BAALC existed in TNBC cells was worthy of exploring. In our study, LRRC75A-AS1 possessing one miR-380–3p binding site was recognized as the sponge of miR-380–3p in TNBC. According to recent reports, LRRC75A-AS1 acted as a negative regulator of vascular calcification^34^. Also, LRRC75A-AS1 repressed colorectal carcinoma cell proliferation and migration^35^. Presently, our work verified that LRRC75A-AS1 functioned as a ceRNA of BAALC in TNBC by sequestering miR-380–3p. Finally, rescue assays proofed that LRRC75A-AS1 facilitated cell proliferation, invasion and EMT processes in TNBC via sponging miR-380–3p to upregulate BAALC.

In conclusion, our study uncovered revealed the contribution of a novel ceRNA network of the LRRC75A-AS1/miR-380–3p/BAALC axis to TNBC development, which might indicate novel biomarkers for TNBC and also shed a new insight into TNBC treatment.

## Supplementary information

Supplement Figure legends

Supplementary Figure 1

Supplementary Figure 2

Supplementary file 1
