# Supplementary material for: Long non-coding RNA LRRC75A-AS1 facilitates triple negative breast cancer cell proliferation and invasion via functioning as a ceRNA to modulate BAALC
Source: Cell Death Dis. 2020 Aug 18;11(8):643. doi: 10.1038/s41419-020-02821-2 (PMC7434919; doi:10.1038/s41419-020-02821-2)
Supplement: Supplementary file 1 — Supplement Figure legends [file 41419_2020_2821_MOESM1_ESM.docx]

**Supplementary Figure 1. A.** qRT-PCR examined expression of BAALC in TNBC tissues and non-tumor tissues. **B.** Kaplan-Meier analysis of TNBC patients with high/low BAALC expression. **C.** Western blot assay measured the protein level of BAALC in TNBC cells (MDA-MB-231, MDA-MB-468, MDA-MB-436, HCC-1937) and in normal mammary epithelial cells (MCF-10A). **D-F.** CCK-8, colony formation and Ki67 immunofluorescence staining assays detected the proliferation ability of MCF-10A, MDA-MB-468 and MDA-MB-436 cells. **G.** Mitochondrial membrane potential was measured by JC-1 assay in MCF-10A, MDA-MB-468 and MDA-MB-436 cells. **H.** Flow cytometry analysis evaluated the apoptosis rate of MCF-10A, MDA-MB-468 and MDA-MB-436 cells. **I.** Caspase-3/8/9 activities were measured in MCF-10A, MDA-MB-468 and MDA-MB-436 cells. **J.** Transwell assay assessed the invasive capacity of MCF-10A, MDA-MB-468 and MDA-MB-436 cells. **K.** Western blot detected the expression of EMT-related proteins in MCF-10A, MDA-MB-468 and MDA-MB-436 cells. **L.** Western blot measured the protein level of BAALC in sh-BAALC#1/2 transfected cells. **M**. CCK-8 assay measured cell viability in sh-BAALC#1/2 transfected cells. Error bars represent the mean ± SD of at least three independent experiments. ^**^P<0.01.

**Supplementary Figure 2. A.** qRT-PCR and western blot measured the mRNA and protein expressions of BAALC in miR-380-3p-mimics transfected cells. **B.** qRT-PCR examined the expression of LRRC75A-AS1 in TNBC tissues and non-tumor tissues. **C.** Kaplan-Meier analysis of the survival time of TNBC patients with high/low LRRC75A-AS1 expression. **D-E.** Subcellular fraction assay and FISH (scale bar = 20μm) assay determined the subcellular location of LRRC75A-AS1 in TNBC cells. **F.** qRT-PCR and western blot measured the expression of LRRC75A-AS1 and BAALC in indicated cells. **G.** RIP assay measured the enrichment of miR-380-3p and BAALC in anti-Ago2 or anti-IgG group in TNBC cells with or without LRRC75A-AS1 silence. **H-I.** qRT-PCR and western blot measured the mRNA and protein expressions of BAALC in indicated TNBC cells. Error bars represent the mean ± SD of at least three independent experiments. ^**^P<0.01.
