# Supplementary figures and images for: Long non-coding RNA LRRC75A-AS1 facilitates triple negative breast cancer cell proliferation and invasion via functioning as a ceRNA to modulate BAALC
Source: Cell Death Dis. 2020 Aug 18;11(8):643. doi: 10.1038/s41419-020-02821-2 (PMC7434919; doi:10.1038/s41419-020-02821-2)

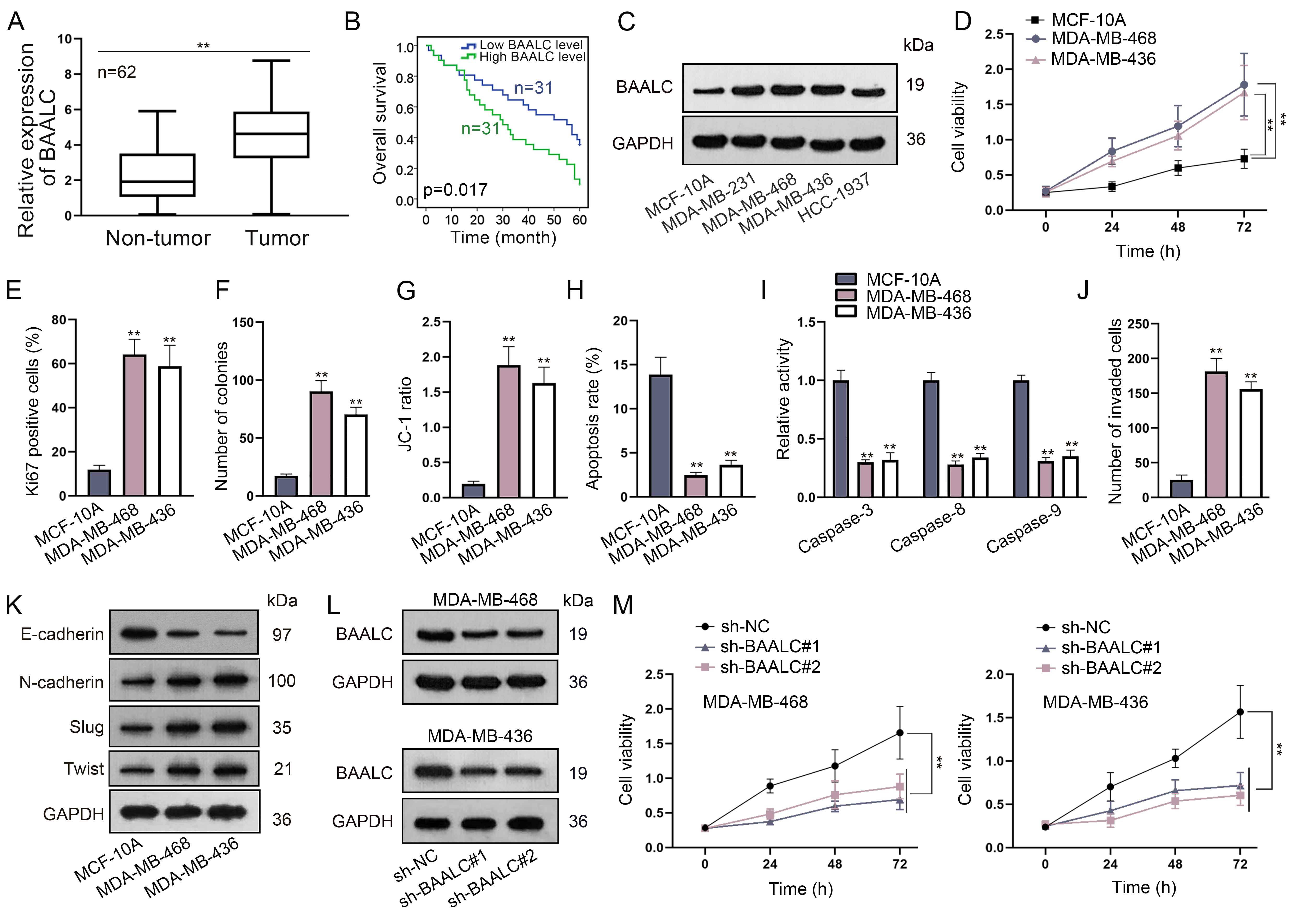

Supplement: Supplementary file 2 — Supplementary Figure 1 [file 41419_2020_2821_MOESM2_ESM.tif]

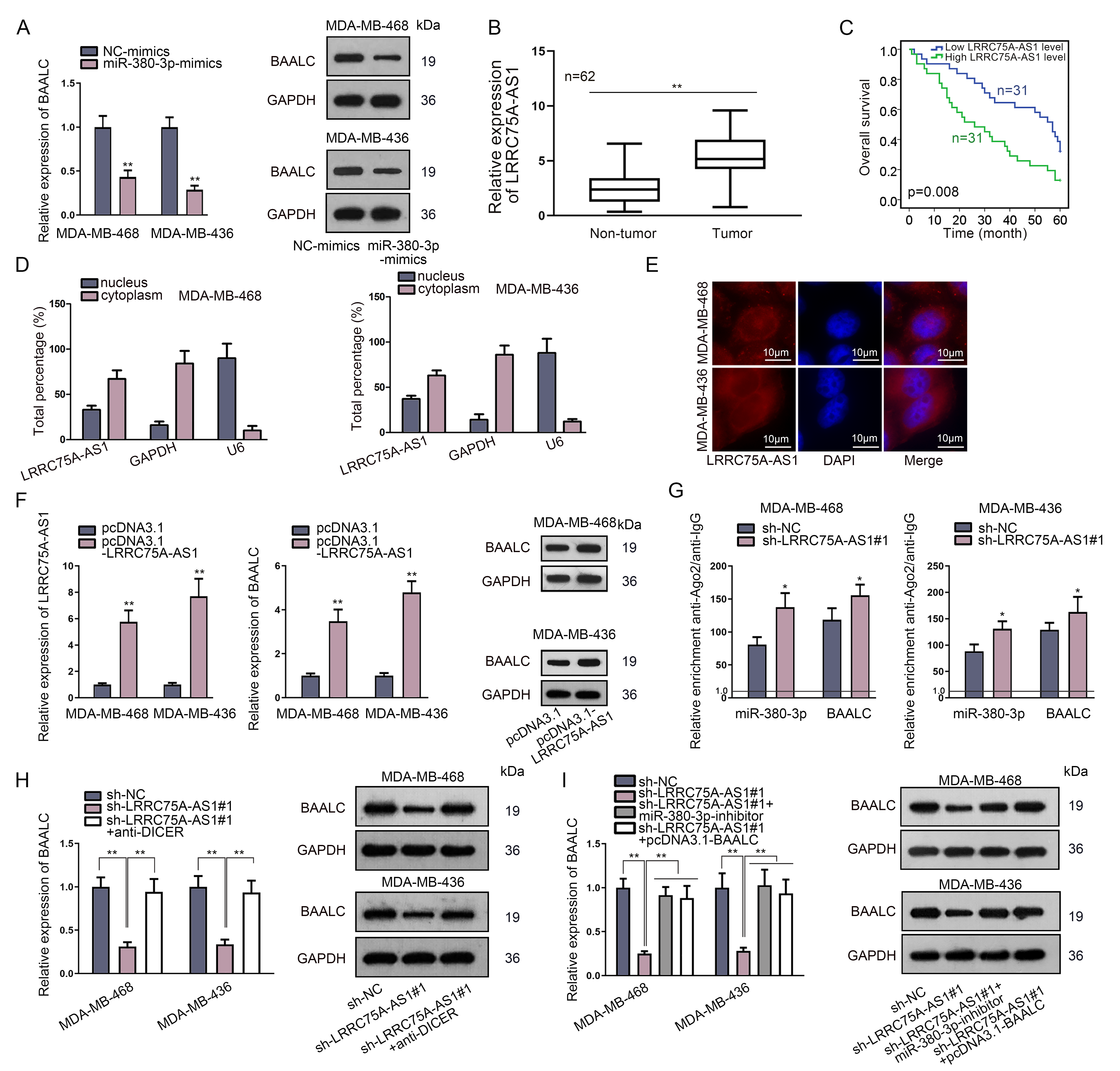

Supplement: Supplementary file 3 — Supplementary Figure 2 [file 41419_2020_2821_MOESM3_ESM.tif]

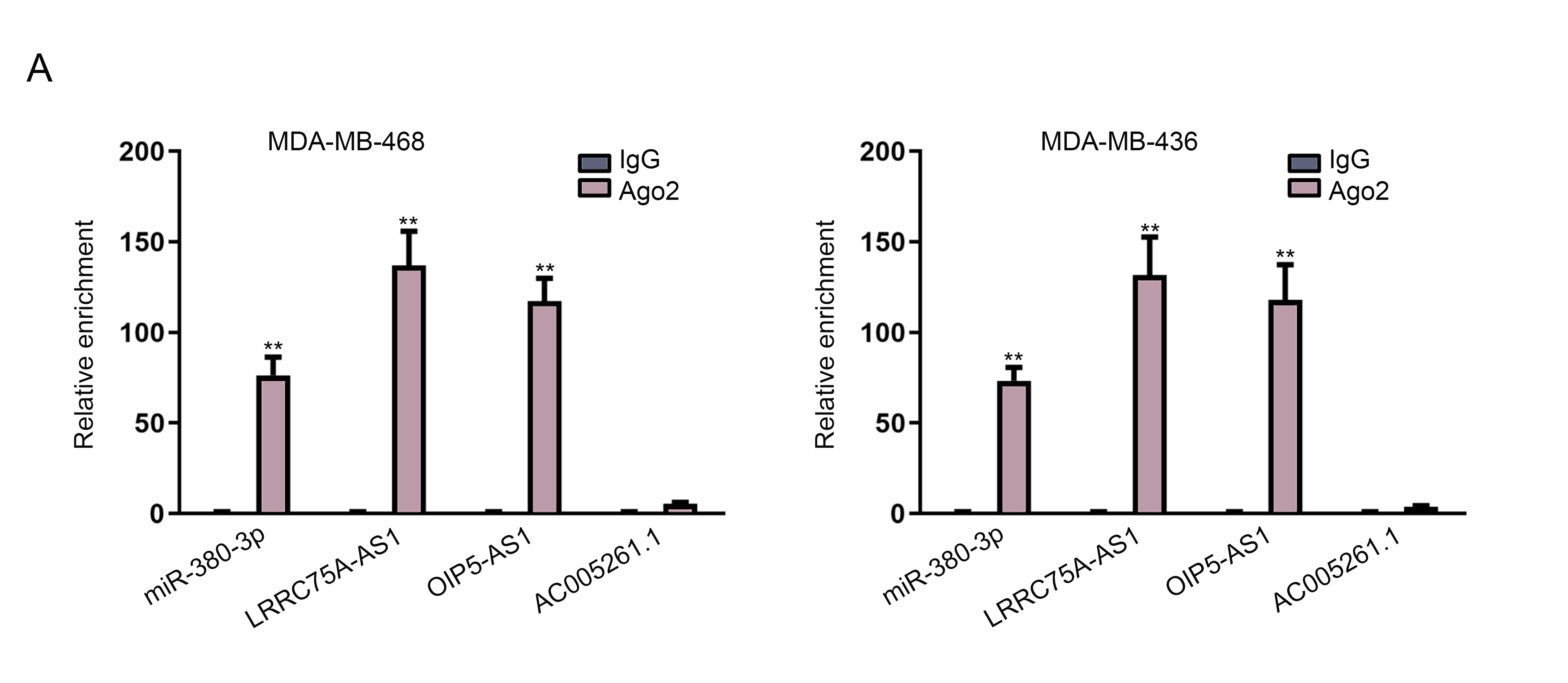

Supplement: Supplementary file 4 — Supplementary file 1 [file 41419_2020_2821_MOESM4_ESM.tif]
